# Highlights of Children with Cancer UK’s Workshop on Drug Delivery in Paediatric Brain Tumours

**DOI:** 10.3332/ecancer.2016.630

**Published:** 2016-03-31

**Authors:** Audrey Nailor, David A Walker, Thomas S Jacques, Kathy E Warren, Henry Brem, Pamela R Kearns, John Greenwood, Jeffrey I Penny, Geoffrey J Pilkington, Angel M Carcaboso, Gudrun Fleischhack, Donald Macarthur, Irene Slavc, Lisethe Meijer, Steven Gill, Stephen Lowis, Dannis G van Vuurden, Monica S Pearl, Steven C Clifford, Sorana Morrissy, Delyan P Ivanov, Kévin Beccaria, Richard J Gilbertson, Karin Straathof, Jordan J Green, Stuart Smith, Ruman Rahman, John-Paul Kilday

**Affiliations:** 1 Cancer Intelligence, 154 Cheltenham Road, Bristol, UK BS6 5RL; 2 Children’s Brain Tumour Research Centre, University of Nottingham, Room EE 1833a Nottingham Children’s Hospital, Queen’s Medical Centre, Nottingham, UK NG7 2UH; 3 UCL Institute of Child Health, 30 Guilford St, London, UK WC1N 1EH; 4 National Cancer Institute, Building 10 - Hatfield CRC, Bethesda, MD 20892–1104; 5 Johns Hopkins University, 600 N Wolfe St, Baltimore, Maryland, USA, 21287; 6 Cancer Research UK Clinical Trials Unit, University of Birmingham, Edgbaston, Birmingham, UK B15 2TT; 7 University College London, 11–43 Bath Street, London, UK EC1V 9EL; 8 University of Manchester, Stopford Building, Oxford Road, Manchester, UK M13 9PT; 9 University of Portsmouth, Winston Churchill Ave, Portsmouth, UK, PO1 2UP; 10 Institut de Recerca Pediatrica, Hospital Sant Joan de Déu, Barcelona, Spain; 11 Essen University Hospital, Paediatrics III, Hufelandstraße 55, Essen, Germany 45147; 12 University Hospital Nottingham, Derby Road, Nottingham, UK NG7 2UH; 13 Medical University of Vienna, Spitalgasse 23, 1090 Vienna, Austria; 14 University Medical Centre Groningen, 1 Hanzeplein, Groningen, The Netherlands, 9713 GZ; 15 Bristol Royal Hospital for Children, Upper Maudlin St, Bristol, UK, BS2 8BJ; 16 VU University Medical Centre, De Boelelaan 1118, Amsterdam, Netherlands, 1081 HZ; 17 Johns Hopkins University School of Medicine, 1800 Orleans Street, Bloomberg Building 7218, Baltimore, Maryland, USA, 21287; 18 Newcastle University, Royal Victoria Infirmary, Queen Victoria Road, Newcastle upon Tyne, UK, NE1 4LP; 19 The Hospital for Sick Children, 555 University Ave, Toronto, Ontario, Canada, ON M5G 1X8; 20 University Hospital Nottingham, Derby Road, Nottingham, UK NG7 2UH; 21 Pitié Salpetriere Hospital, Assistance Publique - Hôpitaux de Paris, 27 Rue Chaligny, Paris, France, 75012; 22 Cambridge Cancer Centre, 120 Cambridge Rd, Great Shelford, Cambridge UK, CB22 5JT; 23 Institute of Child Health, University College London, 30 Guilford Street, London, UK WC1N 1EH; 24 Institute for NanoBioTechnology, Translational Tissue Engineering Center, The Johns Hopkins University School of Medicine, 400 N. Broadway/Smith Building Room 5017, Baltimore, MD 21231; 25 University Hospital Nottingham, Derby Road, Nottingham, UK NG7 2UH; 26 University Hospital Nottingham, Derby Road, Nottingham, UK NG7 2UH; 27 Royal Manchester Children’s Hospital, Oxford Rd, Manchester M13 9WL

**Keywords:** drug delivery, translational medicine, paediatrics, brain tumours, glioma, medulloblastoma, CNS involvement, blood–brain barrier

## Abstract

The first Workshop on Drug Delivery in Paediatric Brain Tumours was hosted in London by the charity Children with Cancer UK. The goals of the workshop were to break down the barriers to treating central nervous system (CNS) tumours in children, leading to new collaborations and further innovations in this under-represented and emotive field. These barriers include the physical delivery challenges presented by the blood–brain barrier, the underpinning reasons for the intractability of CNS cancers, and the practical difficulties of delivering cancer treatment to the brains of children. Novel techniques for overcoming these problems were discussed, new models brought forth, and experiences compared.

## Introduction

In February 2016, a unique Workshop on Drug Delivery in Paediatric Brain Tumours took place at the Royal College of Physicians, London, UK.

The workshop was initiated and funded by Children with Cancer UK, a charity dedicated to fighting childhood cancer through research and welfare projects. Although the title of the workshop referred to paediatric brain tumours, the scope of the talks included all central nervous system (CNS) tumours, and techniques and discoveries developed in CNS tumours in adults.

Cancer remains the most common medical cause of death beyond infancy in children and young people in the UK. After leukaemia, cancer of the CNS is the next most common form of childhood cancer [1]. Children with Cancer UK was founded in 1988 and originally focused on childhood leukaemia; in 2011, the charity broadened its remit to include all childhood cancers. The workshop formed part of the three-year Brain Tumour Initiative, which was launched in 2014.

Following the workshop, Children with Cancer UK is calling for project grant applications focused on the delivery of therapy in paediatric CNS tumours.

With 27 speakers and over 90 participants from every corner of the globe, this workshop brought together leading experts in the fields of drug delivery, brain tumours, clinical design, paediatric care, and other related fields. The goals of the workshop were to pool and share the knowledge and experience of these disciplines, to lay out new research plans and collaborations, to bridge gaps between fields of research and clinical work, and to innovate solutions to overcome the barriers to treatment inherent in brain tumours in young people.

These barriers include physical challenges presented by the blood–brain barrier, the intractability of CNS tumours, and the practical difficulties of delivering treatment to children.

The event was chaired by Professor David Walker, Professor of Paediatric Oncology, of the University of Nottingham, Nottingham, UK. Dr Walker is the chairman of Children with Cancer UK’s scientific advisory panel.

‘The time is right to think carefully about how we can tackle this CNS drug delivery challenge through innovation, experimentation and translation so that new systems are in trial within five years,’ Dr Walker explained.

## Setting the scene

‘Worldwide, 260,000 children develop cancer each year,’ Professor David Walker explained in his introductory lecture, opening the workshop and describing the context of this unique treatment landscape. ‘Of these, between 30,000 and 40,000 children have CNS tumours.’

After leukaemia, CNS tumours are the most common form of childhood cancer in the UK. They are uniquely difficult to treat. At the forefront of these challenges, the blood–brain barrier disallows the transmission of large molecules, such as cancer drugs, into the tissues of the brain. Brain and CNS tumours have a particularly grim outlook. Dr Walker also noted the duty of care that oncologists have to child patients. Child development must be a key concern with cancer treatment; in particular, cranial irradiation is shown to have an adverse effect on IQ in children, while cranial and craniospinal irradiation are shown to inhibit physical growth. Cancer treatment can lead to CNS damage, stunted growth and disability.

Dr Walker proposed a development framework to address these problems, calling it ‘Hatch, Match, Despatch and Deliver’.

He reminded the participants of the goals of the workshop—namely, to sow the seeds of inspiration and collaboration between disciplines and participants.

He was followed by Dr Thomas Jacques of the University College London Institute of Child Health, London, UK, who provided a primer on the pathology of childhood brain tumours. ‘Why do children die of brain tumours?’

Dr Jacques brought a pathologist’s perspective to this problem. He explained the necessity of accurately predicting the child’s response to cancer treatment—not only because a child has a lifetime of potential ahead of them to preserve, but also because, as Dr Walker said, sustained treatment of the CNS can lead to long-term consequences. Predicting treatment response through pathology is necessary to tip this delicate balance in the child’s favour.

Historically, pathology relied on the microscopic examination of tissue samples, but advances in the field mean that modern pathologists can integrate molecular biology and genomics into a broader report.

Why do children die of brain tumours? Dr Jacques explained that the clue to answering this problem is ‘understanding the tumour at the point that it kills you.’ Naturally, this is the point at which the tumour is no longer responsive to treatment—often because it has evolved resistance to the regimen. The key, then, is studying the tumour at that point.

This is another area in which the treatment of children poses a psychological challenge; Dr Jacques estimates that contrary to common societal beliefs, 89% of parents in this situation have found autopsies to be helpful. Not only may autopsies serve to provide closure to bereaved parents, they are necessary to advance this understanding of the tumour’s pathology. Oncology professionals in other disciplines might find this useful to consider when advising families.

‘It doesn’t matter how good the drug is—if you don’t deliver it to the tumour, the treatment won’t work,’ said Dr Kathy Warren of the National Cancer Institute, Washington, USA as she introduced her talk. She explained that in cancer research generally, we are moving beyond the era of relying heavily on cytotoxic drugs, and into a more nuanced era of genomics and targeted therapies; however, these advances have not yet benefited children with CNS tumours, partially due to the challenge of the blood–brain barrier.

Dr Warren identified three main pathways to circumvent the BBB: penetration, disruption, bypass, and various combinations thereof. Each of these pathways has limitations.

Dr Warren noted that even drugs that are designed to penetrate the blood–brain barrier are diffused by it, resulting in variable delivery. Thus, methods to circumvent or breach the BBB may increase precision. Drugs such as lobradamil, a bradykinin agonist, temporarily disrupt the cohesion of the BBB, allowing combination therapy within a limited window. Recently, focused ultrasound has shown the ability to physically disrupt the barrier. Lobradamil, which Dr Warren has found to increase uptake, may be a useful agent in combination therapies.

The BBB can be physically bypassed, as well. Dr Warren noted the success of intratumoural delivery—particularly Gliadel wafers—which remain one of the most successful treatment methods for brain tumours, but do not address the treatment of diffuse tumours. Likewise, intrathecal delivery physically bypasses the BBB and will also be covered later in this report—and although it dodges the specific problem of the BBB, it faces the issue of a similar barrier between the cerebrospinal fluid (CSF) and the brain, limiting penetrance.

Dr Warren noted that many of our current paradigms of drug development are remnants from the era of cytotoxic drugs, operating under the assumption that the maximum tolerable dose of drugs delivered at the shortest tolerable interval will be the most effective. In the case of paediatric CNS tumours, this may not be a valid assumption. Dr Warren stated that identifying the *minimum* effective drug exposure, delivered precisely in a targeted manner, may be a more rational method of drug development. She supported this by noting that the paradigm of predicting maximum doses in adults is not always appropriate for child patients.

Dr Warren’s arguments were very persuasive, and her evidence certainly supported the paradigm of minimal therapies delivered in a precise manner.

Many participants had been eagerly anticipating the lecture by Professor Henry Brem of Johns Hopkins University, Baltimore, USA, and he did not disappoint, providing a history of the field. Professor Brem is certainly a doyen of the field of brain tumour treatment, particularly notable for his work on Gliadel. However, it was his personal perspective of the field that made his first talk of the workshop particularly notable. Professor Brem’s continuity of experience has given him a unique insight on the development of brain tumour treatment—In 1984, when he began his career, he was strongly advised by his superiors not to start his academic career on brain tumours, as it was so dismal a field!

In 1984, patients with brain tumours had a particularly grim outlook; the FDA had not approved any new therapy for 20 years, and the median survival was nine months. Today, with agents such as Gliadel, temodar and Avastin, the median survival has doubled.

Gliadel is a biodegradable polymer (polifeprosan 20) shaped into a wafer and impregnated with carmustine. To deliver this treatment, the brain tumour is first physically excised with surgery, creating a cavity in the brain. This cavity is then lined with Gliadel wafers, which deliver a slow, consistent release of carmustine. The particular attractions of this treatment are that it bypasses the BBB entirely; it delivers treatment in a very targeted area; and it has been shown to delay recurrence in the excision area. In combination with radiation and oral Temozolomide (as well as the excision and surgery needed to place the wafers) the use of Gliadel has led to an increase in median survival to 18–21 months.

But when Professor Brem began his work on Gliadel, he met with over a decade’s worth of resistance. ‘They told me it was a nice idea, but would never work,’ he said. Early concerns were that the polymer itself would be toxic, that the drug would not be released properly, and that it was an inefficient delivery system. Other concerns, such as patient resistance, the difficulty of organising trials, and the possibility of infection at the surgical site provided challenges in their time. Future speakers in the workshop elaborated upon these various complications in greater detail.

What are Professor Brem’s feelings about the current treatment landscape?

‘The opportunities have never been greater,’ he said, ‘But there will always be barriers.’

Professor Pamela Kearns of the Cancer Research UK Clinical Trials Unit (CRCTU), University of Birmingham, Birmingham, UK described the current landscape of clinical trials in paediatric CNS tumours in the UK. When attending multidisciplinary workshops such as this one, she noted, participants often wonder, ‘where are the outcomes of all this innovation?’ The answer, of course, is in the still somewhat-mystifying landscape of clinical trials.

Professor Kearns informed the audience that at the time of the workshop, the only clinical trial on paediatric CNS tumours open in the UK was SIOP-E’s phase III intracranial germ cell study which, like the majority of clinical trials in this field, is international. However, she stated that the UK is well-positioned to lead clinical trials in this field, with good support including the CR UK core-funded CRCTU at the University of Birmingham, and that there are opportunities for more development.

CNS tumours are identified as a priority cancer of unmet need in the current CRUK research strategy. The CRCTU will open five trials for paediatric patients with CNS tumours in the next twelve months.

One challenge that collaborators must rise to meet is that of ‘standards of care,’ which are not standardised across international collaborations. The CRCTU is planning to undertake systemic reviews of treatments to evaluate the current level of evidence to support best practice in paediatric CNS tumours, with the hopes of informing future trial questions and designs.

The timely delivery of trials is a challenge. Focusing on ways to reduce trial development times and increase accessibility of trials to all children and young adults will go a long way towards increasing the effectiveness of conducting clinical trials in these patient populations. Professor Kearns noted that it is important to keep sight of the intended outcomes—that improved clinical trial design will improve outcomes for children and young people with cancer.

‘Trials are labour intensive,’ she said, ‘But without them, we can’t change clinical practise.’ She noted the ongoing importance of international collaboration, and her hopes that researchers will be inspired to seek such opportunities.

### Crossing the blood–brain barrier

Professor John Greenwood of the University College London, London, UK was asked by the workshop leader to provide a general overview of the blood–brain barrier. For a multidisciplinary gathering of this nature, this thorough review ensured that participants had a consistent basic understanding of the BBB. The endothelial cells of the BBB are well-known for their barrier properties. Under normal circumstances, this barrier prevents large molecules, immune cells, and other particles from accessing the brain tissue. The overall neurovascular unit is a dynamic entity. Some brain cancers disrupt the integrity of the BBB; likewise, the BBB limits the spread of many brain cancers.

Professor Greenwood explained that dysfunction of the BBB can both help and hinder brain cancer treatment. When intact, the BBB limits the penetrance of therapeutic agents, as observed in previous talks.

What makes a drug able to penetrate the blood–brain barrier? Dr Jeffrey Penny of the University of Manchester explained that molecules cannot pass the ‘gatekeeper’ of the BBB if they are too highly charged, too large and not lipid soluble.

The two main routes of crossing the BBB are the paracellular route, which is impeded by tight junction complexes, and the transcellular root, which is impeded by transporter proteins. The most successful drugs that approach the paracellular route are lipophilic, able to cross the BBB by diffusion. The brain’s mechanism to remove toxins can pump out cancer drugs that attempt to enter through transporter-mediated uptake, so the most effective compounds for entering the brain by this route often resemble natural compounds.

Drugs that disrupt the barrier of the BBB, such as valspodar, can be used in combination with agents such as paclitaxel to increase uptake. Cyclosporin A, which has been shown to knock out efflux transport in other tissues, also increases bioavailability of drugs administered in combination.

In particular, Dr Penny noted that it is important to *predict* how the BBB will affect drug handling and drug delivery. This will lead to more efficient drug development, as well as improving the efficacy of existing therapeutic agents. He has developed an *in vitro* model of porcine brain endothelial cells which is showing promise as a model of the BBB.

Professor Geoff Pilkington of the University of Portsmouth, Portsmouth, UK introduced more *in vitro* models of the BBB in the following talk. He noted that a difficulty with replicating the BBB *in vitro* is the heterogeneity of the complex itself—involving astrocytes, brain endothelial cells, and pericytes. In co-culture situations, this can be challenging, as different cell types have different needs regarding growth media and passage. Tri-cultures are used when adding pericytes.

‘Is the pericyte a black sheep, or a Scarlet Pimpernel?’ Professor Pilkington asked humorously. Long left out of research models in favour of easily cultured endothelial cells, pericytes are now more widely appreciated. Likewise, co-culture and 3D culture methods are increasingly recognised as providing a more realistic *in vitro* model. Often, mixes of cell lines from various animals are used; Professor Pilkington’s models are particularly interesting as his co-cultured cell models are all of human origin.

These models appear to be remarkably successful, with ‘hitherto unreportedly high’ levels of transendothelial resistance (TEER), implying that the co-cultured cells had formed tight junctions, reflecting the physiology of the BBB.

Professor Pilkington’s models show promise for elucidating the mechanisms of medulloblastoma metastasis—is the spread via leptomeningeal or vascular routes? Finally, Professor Pilkington has had some success in penetrating his BBB models with nanoparticles. A particular challenge will be modifying these models for use in paediatric brain tumours.

Dr Angel Montero Carcaboso of the Hospital Sant Joan de Déu, Barcelona, Spain then provided a lectura on why CNS drug penetration matters and how to address it. Some drugs accumulate in cerebrospinal fluid (CSF) more than in the brain, or do not penetrate at high concentrations in the brain parenchyma. Among paediatric brain tumours, diffuse intrinsic pontine glioma (DIPG) is likely to be one of the most difficult to tackle. Tactics such as surgery and Gliadel implantation are not feasible in diffuse tumours, and the BBB is known to impede the systemic delivery of treatment, possibly resulting in low concentrations of drugs across diffuse tumours.

Key questions include whether chemotherapy agents are successful in penetrating the BBB to treat DIPG, and whether drug concentrations in the CSF are indicative of drug distribution in the brain, which is currently unknown. Further, irinotecan was found to have promising activity *in vitro*, but this did not translate to the clinic. A Phase II trial of irinotecan in combination with cisplatin failed to show therapeutic activity in DIPG patients at HSJD.

In the hopes of answering these questions and understanding this trial failure, Dr Carcaboso developed preclinical DIPG models in which xenografts of patient tumour stem cells are established in mice. The mice were treated with systemic administration of irinotecan. Examination of mouse tissues showed that campthotecin SN-38, the active metabolite of irinotecan, failed to penetrate the CNS at an active concentration. Further, there was no benefit in survival upon systemic irinotecan-cisplatin treatments; in this, the model replicated the human trial. Imaging with systemic fluorescein confirmed that the BBB had remarkably conserved function in the animal model. This implies that the BBB impedes the penetration of the treatment across these diffuse tumours.

Dr Carcaboso concluded that accurate preclinical models are necessary to develop treatments for brain tumours. ‘Tumour models need to recapitulate the genetics and functional characteristics of the patient tumour,’ he said, as part of this convincing argument.

### Intra-CSF therapies

What can be learned from previous intra-CSF studies in childhood malignancies? Professor Gudrun Fleischhack of Essen University Hospital, Essen, Germany presented a talk on the use of intrathecal or intra-CSF therapy, in which therapeutic agents are injected directly into the cerebrospinal fluid. Intrathecal delivery of methotrexate has shown great success in childhood acute lymphoblastic leukaemia (ALL) in the CNS. Because this method of delivery bypasses the blood–brain barrier, it is an attractive treatment option for brain and other CNS tumours, and may overcome treatment challenges of diffuse tumours. Previous studies in intrathecal administration show that it is generally well tolerated, with mostly mild adverse effects.

Methotrexate is an attractive drug for intrathecal therapy, as it possesses many of the pharmacokinetic requirements for effective administration. However, slow-growing solid tumours may be less responsive to this cycle-specific drug. Etoposide, topotecan and lipsomal cytarabine have shown promise, and new immunotherapeutic agents are under evaluation, but Professor Fleischhack says that there is no ‘ideal drug’ that possesses all the desirable attributes for this therapy.

Thus, the hunt must continue for drugs that are both effective in solid tumours and appropriate for intrathecal administration. Professor Fleischhack highlighted this as an area for potential international collaboration.

Therefore, Professor Fleischhack concludes, there is compelling need for novel agents, refinement in drug delivery systems and improvement of trial designs to test the efficacy of intra-CSF therapies, especially in solid tumours.

Professor David Walker returned to deliver a lecture on the topic of intrathecal chemotherapy treatment for medulloblastoma in children. He noted that intrathecal therapy spares the child’s brain, offering an attractive alternative to the ‘unacceptably neurotoxic’ programme of craniospinal radiotherapy. He agreed with Professor Fleischhack that the lack of suitable drugs to deliver via this method is a key challenge. The delivery method is promising, he noted, but suitable drugs need to be explored. This is part of the ‘Hatch’ phase of his proposed drug development scheme—identifying the right drugs for the problem.

So what is the ideal intrathecal drug? Dr Walker’s list of ideal characteristics, combined from literature review and pharmacokinetic analysis, state that it should be non-irritant, should ionise at the pH levels of cerebrospinal fluid, should be hydrophilic, and should be a large molecule. There should not be a carrier system that removes it from the CSF, and ideally it should not be specific to the cell cycle, as Professor Fleischhack noted. It should exhibit low toxicity by the systemic route and the intrathecal route, with rapid distribution through CSF compartments and a slow rate of clearance from the CSF. It should be available in injectable formulation, and should either be active within the CSF, or possibly activated by enzymatic agents within the CSF itself.

Based on this list of characteristics, Dr Walker identified 126 existing candidate drugs with potential utility for intrathecal administration in brain and CNS tumours. Of these, the vast majority did not meet the needs of the application. Of the 27 drugs remaining, about half are eligible for further clinical investigation (12), and the rest are suitable for pre-clinical investigation.

Donald Macarthur of the University Hospital Nottingham, Nottingham, UK followed with a discussion on the clinical aspects of intra-CSF therapy, addressing patient selection, the practicalities of CSF access, and complications.

Macarthur prefers delivering intrathecal chemotherapy through an Ommaya reservoir, although lumbar ports can also be installed. He discussed his experience with the clinical administration of intrathecal chemotherapy in a cohort of twenty paediatric patients.

Appropriate patients should be selected based on the anatomical distribution of their disease. Their goals should be considered—do they have curative or palliative goals?

He paid particular attention to the practicalities of CSF access and port management, which are a key consideration for parents of young children. There have been child deaths due to misapplied intrathecal chemotherapy, and parents may fear these risks. Recent advances in lumbar port design have limited these risks, following a 2009 recommendation to make lumbar port connections incompatible with standard Luer syringes. Other ways to reduce complications and improve safety include meticulous checking and training. Tattoos can be used, similar to radiotherapy tattoos, to ensure consistency of application—they can even be designed for aesthetic appeal.

Complications can include infection, sepsis, wound leakage, and migration of ports and shunts. But sixty percent of his patients had event-free treatment. Six of his twenty patients are still alive.

Macarthur noted that intrathecal therapy is useful for the palliation of leptomeningeal disease, that it could form part of multimodal treatment, and that it may help younger patients avoid the burden of radiotherapy.

Professor Irene Slavc of the Medical University of Vienna, Vienna, Austria presented on the DepoCyte sustained release system for intra-CSF delivery, which her colleague Professor Fleischhack had touched on previously. The parent drug cytarabine, she noted, is a S-phase specific antineoplastic compound mainly used in the treatment of AML, ALL and lymphoma. I.th cytarabine is the second most widely used drug for treatment of meningeal neoplasia. The terminal half-life of parent drug cytarabine, however, is only 3.4 hours because the clearance from the CSF is similar to the CSF bulk flow rate of 0.24 mL/min. Solid tumours have a lower proliferation rate than hematologic malignancies and therefore duration of exposure of neoplastic cells to cytotoxic drug concentrations is important for efficacy. Liposomal cytarabine was developed in the 1980s and is a slow-release formulation in which cytarabine is encapsulated in microscopic particles. Pharmacokinetic studies of intraventricular administration of the slow release formulation liposomal cytarabine by her group and others have shown that cytotoxic drug concentrations are maintained for at least 8 days. Liposomal cytarabine is generally well tolerated if dexamethasone is administered concomitantly to prevent arachnoiditis. In its sustained-release form it is called DepoCyte.

As Dr Walker noted in his previous lecture, the makeup of the CSF itself should be considered when administering drugs. Cytidine deaminase, the enzyme that catalyses the metabolism of cytarabine, is present in low levels in the CSF, meaning that the drug is cleared more slowly and remains in the system for longer. As cytarabine is cell-cycle specific, it must be applied for a longer duration of exposure, so this property makes it particularly suitable to treat haematological malignancies in the CNS. However, in the case of solid tumours which proliferate slowly, this is a setback—cytarabine would have to be administered continuously to be effective. Thus, the slow-release form of cytarabine (DepoCyte) is an attractive choice for intrathecal chemotherapy. Professor Slavc found that it is safe and well-tolerated if given concomitantly with dexamethasone.

The terminal half-life of the drug is shorter in children, particularly children under the age of three. Treatment-related complications are present in up to a quarter of adult patients. Professor Slavc feels that the development of slow-release treatments is encouraging, and heading in the right direction.

Dr Lisethe Meijer of Beatrix Children’s Hospital of the University Medical Centre Groningen, Groningen, The Netherlands and the Children’s Brain Tumour Research Centre, University of Nottingham, UK explained how these preclinical developments translate to clinical trial design. She introduced a Phase I feasibility, safety and tolerability study on continuous INTRa-cerebrospinal fluid infusion of EtoPosIDe (INTREPID). The INTREPID trial will investigate dose escalation and duration of infusion in children and adolescents with leptomeningeal metastases of solid tumours. Its aim is to deliver a steady state of continuous etoposide infusion to these tumours in young patients.

Leptomeningeal metastases, a complication of solid tumours, have dismal survival outcomes and are difficult to treat. For reasons discussed throughout the other talks, systemic and radiotherapy treatments are not ideal, and intrathecal delivery is an attractive option.

To determine the ideal dose, Dr Meijer and colleagues tested etoposide *in vitro* in both 2D and 3D cell cultures. As other researchers at the workshop had discussed, there was a significant difference in results between these two methods. The 3D culture most faithfully replicated findings *in vivo*. This evidence supports the belief that drug development should pursue 3D cell culture.

She also presented the LeptoMenIngealMetastases RADiOlogicalResponse (MIRADOR) Criteria. LM is observable through imaging before clinical signs present. Validated radiological criteria are needed to accurately evaluate responses in INTREPID and future clinical studies. These criteria are in development, and Dr Meijer stated her interest in receiving feedback.

In the discussion after the session, Professor David Walker reminded participants that ‘visiting the hospital every day for treatment is not compatible with normal daily life.’ Where possible, intrathecal therapies requiring less frequent hospital visits should be developed.

### Tackling DIPG by enhanced drug delivery techniques

‘To improve survival, we must think outside the box,’ said Dr Dannis van Vuurden of the VU University Medical Centre, Amsterdam, The Netherlands. He presented an overview of convection-enhanced delivery trials and perspectives for paediatric neuro-oncology. Convection-enhanced delivery (CED) involves the implantation of micro-catheters, allowing drug to be directly infused into the brain tissue at a slow consistent rate.

‘Not all tumours are suitable for convection-enhanced delivery,’ he told *e*cancer in a later interview. Not all drugs are suitable either—there are certain properties of the drugs, such as charge, lipophilicity and whether these drugs are substrates for ABC-transporters (drug pumps), that influence how suitable these are for CED. So there are some limitations to the technique, which need further investigations and collaborations with pharmaceutical companies.

Small studies examining this technique have shown promise, but larger studies are needed. Children may benefit more than adults.

‘I think the take home message is that we need to think of incorporating new technologies in current-day treatments to find a cure for paediatric brain tumours. These are big words, but I’m definitely very hopeful that this will happen in the near future.’

Professor Steven Gill of the Bristol Royal Hospital for Children, Bristol UK presented his early clinical experience of convection-enhanced delivery of drugs for paediatric DIPG. As previous participants noted, these high-grade diffuse tumours have a dismal outlook, and are difficult to treat; they are not resectable, chemotherapy does not lead to improved survival, and radiotherapy is the mainstay of treatment. Professor Gill reminded participants that patients with DIPG have a median survival of nine months, and 90% of children die within 18 months of diagnosis.

Professor Gill has developed a novel method of CED involving a unique implantable drug delivery system for intermittent infusions directly into the brain parenchyma. The device comprises four catheters which are delivered to deep brain targets with robotic assistance. The material of the indwelling micro-catheters prevents them from blocking between infusions, and its step design reduces reflux and facilitates drug delivery by bulk flow over clinically relevant brain volumes. The catheters are each connected to separate channels in a septum-sealed, transcutaneous and skull-mounted port. Drug can be repeatedly delivered by external pumps after attaching an administration set to the port.

Fascinatingly, Professor Gill and his research group studied horned mammals to design the infection-resistant catheter ports. The transcutaneous element of the bone anchored port would potentially be vulnerable to infection; however, by removing the sub-dermal fatty tissue immediately around the port and providing a micro-textured surface on the port’s tissue interface that facilitates adhesion, the infection-resistant mechanisms of an animal horn are mimicked.

Professor Gill used combined magnetic resonance imaging (MRI) and computerised tomography (CT) scans to plan the positioning of the catheter arrays, which are delivered with robotic assistance. ‘We must plan every move of the surgery before we begin,’ he said.

He carried out this work on a cohort of six children at the Children’s Hospital, Bristol where Carboplatin delivered by CED has been approved for compassionate use. Professor Gill’s high flow, low pressure catheters allow several millilitres of fluid to be administered per day into the pons—up to 12 mL per day in the case of one five-year-old female patient.

In summary, he found that carboplatin delivered intermittently by this method controlled the DIPG in his six young patients, and extended their survival. A trial will commence in mid to late 2016. He encouraged fellow participants to consider the use of CED at the point of diagnosis.

Dr Stephen Lowis of the Bristol Royal Hospital for Children, Bristol, UK discussed the selection of drugs and their pharmacokinetics for use in CED. ‘Our experience with CED is limited, and the measurement of normal pharmacokinetic parameters are unusually difficult,’ he said. Instead, we must look at principles, such as pharmaceutical factors (is the drug soluble in artificial CSF? What is its ionic charge?), characteristics of the delivery system (what is the catheter size and design? Are there concerns of tissue damage?), characteristics of the brain and tumour tissue (is oedema present? Are there cystic spaces?), characteristics of the vascular system (are new vessels permeable? Is the BBB intact?) and rate of delivery of the convected drug.

With a shared understanding, Dr Lowis hopes that fellow researchers will be able to develop better strategies, bringing suitable drugs into clinical practise.

Dr Monica Pearl of the Johns Hopkins University School of Medicine, Baltimore, Maryland, USA offered a slightly different angle in her talk on intra-arterial chemotherapy methods, which involve placing a microcatheter in a related artery. Her particular experience is with intra-arterial chemotherapy for retinoblastoma.

‘The eye is a good target for this therapy, as it is an end organ for the ophthalmic artery, allowing for super-selective drug delivery,’ she said. This treatment has emerged in the United States in the past decade and is feasible, effective and minimally invasive. Given this success, Dr Pearl suggests that it could be considered for DIPG.

She introduced her pilot study for bringing these techniques to DIPG, by catheterising the basilar artery and delivering melphalan through it.

Early refinements on rabbits allowed Dr Pearl’s team to visualise drug delivery, predict BBB disruption and perfusion territory, titrate the biodistribution, and design a future tumour model.

In a concurrent project, Dr Pearl has developed an MRI-guided platform for intraarterial delivery after focal BBB disruption. The MRI-guided approach allows them to pre-define the area for BBB disruption, and to track the trans-catheter perfusion of the drug.

‘While our current methodology utlilises the chemotherapy drug melphalan, this delivery strategy can be applied to a variety of therapeutic agents,’ she explained.

### Models of medulloblastoma for drug delivery

Professor Steve Clifford of Newcastle University, Newcastle upon Tyne, Newcastle, UK explained the challenges of medulloblastoma relapse. Relapse following multi-modal therapy is the most adverse event in medulloblastoma, he noted, with over 90% of relapsing patients dying, accounting for about 10% of childhood cancer deaths. Medulloblastoma is heterogeneous at diagnosis and comprises of four distinct molecular subtypes; WNT-medulloblastoma is the most readily cured, while Group 3 has the worst prognosis. The clinical relevance of these subtypes in relation to relapse is currently unknown. Further complex features of medulloblastoma include TP53 pathway status and MYC/MYCN amplification.

To examine how medulloblastoma changes at the point of relapse, Professor Clifford took samples of 29 recurrent tumours paired with tumour samples taken at diagnosis.

He found that a common feature of relapsed tumours were MYC gene family amplifications and TP53 pathway defects, and that combined MYC-p53 defects predicted rapid progression to death. With this evidence of MYC-p53 interactions, he turned his attention to a novel mouse model. The spontaneous development of *Trp53*-inactivating mutations was also noted in a transgenic mouse model of MYCN-driven medulloblastoma. The mouse model was able to replicate the clinicopathological characteristics of TP53-MYC relapsed tumours.

He concluded that the molecular, pathological and clinical characteristics of medulloblastoma are altered at relapse. This underpins one of the challenges of treatment—that it is often determined by findings from the initial biopsy, which do not accurately reflect the characteristics of the relapsed tumour. Harkening back to Dr Jacques’ earlier statement—that we must understand the tumour at the point that it is lethal—Professor Clifford stated that a biopsy at relapse is essential, and that ‘we must develop strategies at relapse that are not dominated by the initial biopsy.’

Dr Sorana Morrissy of the Hospital for Sick Children, Toronto, Ontario, Canada offered a much-needed evolutionary perspective on the development of recurrence. ‘Medulloblastoma is really four different diseases,’ she said, with a heterogeneous mixture of cells present in the tumours. The evolution of cancer resistance parallels antibiotic resistance; while treatment can destroy populations of cells, this selects cells that are resistant to treatment, giving rise to lineages that proliferate and hasten relapse.

Dr Morrissy treated a transposon-driven, functional genomic model model of medulloblastoma with surgery followed by CT-guided craniospinal radiation. Some mice were cured or exhibited prolonged survival, but many relapsed, and Dr Morrissy found that the genetic makeup of the tumour at relapse showed very little overlap with that of the tumour at diagnosis. Thus, the treatment changed the genetic profile of the tumour.

In both the mouse model and human studies, the dominant clone at recurrence arose through selection of a pre-existing minor clone that had been present at diagnosis. In fact, Dr Morrissy says, recurrence can be derived from a single lineage in the primary tumour.

Looking at the extensive genetic divergence between the primary and recurrent tumour, Dr Morrissy found that cancer treatment imposes a population bottleneck, analogous to macroevolutionary selection. Further, since therapy naturally eliminates the lineages that are susceptible to treatment, the population that emerges at recurrence is selected by resistance to the treatment. Dr Morrissy used the metaphor of a ‘fruit salad tree,’ a grafting technique in which multiple types of citrus fruits can be grown on one tree; if a person snipped off every lime on the tree, the tree could quickly repopulate itself with oranges and lemons. The heterogeneous nature of medulloblastoma tumours means that even if targeted treatment ‘snips off’ every cell in a lineage, recurrence can begin from the remaining lineages.

‘Targeted therapy is unlikely to be effective in the absence of a target,’ she explained. ‘Therefore, our results offer a simple explanation for the failure of prior clinical trials of targeted therapy to address relapsed medulloblastoma.’

Dr Morrissy’s lecture was an extremely convincing argument for the necessity of appreciating the evolutionary perspective when understanding cancer resistance and relapse, particularly in such dynamic populations of tumours.

Dr Delyan Invanov of the University of Nottingham, Nottingham, UK introduced preclinical models of medulloblastoma in the next presentation, humorously quoting statistician George E. P. Box—‘All models are wrong, but some are useful.’

‘What makes a model good?’ he asked, noting that ‘useful’ models demonstrate physiological relevance and therapeutic prediction.

‘It isn’t just about cell lines, it’s how you are growing them,’ he added, critiquing the somewhat simplistic nature of monolayers as useful for screening drug libraries but limited in clinical relevance. As other speakers had supported the use of 3D cell culture over 2D monolayers, the audience was receptive. In the case of medulloblastoma tumours, which are heterogenous, Dr Ivanov argued that co-cultured cell lines are necessary. Further, 3D co-culture introduces important characteristics such as physiological gradients and cell-cell contact. Dr Ivanov suggested that this ‘new generation’ of *in vitro* models is the best way forward.

Next, Dr Ivanov argued that while *in vitro* studies are attractive and cost-effective, animal models more faithfully replicate drug-target engagement and more closely approach physiological relevance.

‘Ultimately, we need a large pool of well-characterised, patient-matched models that can capture the heterogeneity of medulloblastoma,’ he explained.

He also suggested that models should be designed with utility in mind, hoping to fill existing gaps—*in vitro* models for Group 3 medulloblastoma are perhaps over-represented, while there are few models for Group 4 and WNT medulloblastoma. Thorough literature review, including systematic reviews and meta-analysis, could lead to development opportunities.

### Novel technologies for blood–brain barrier penetration

Returning to the challenges presented by the BBB, Dr Kévin Beccaria outlined his work on localised BBB disruption using pulsed ultrasound. Transcranial focused ultrasound has been already been used in humans for treatment of neurological disorders such as Parkinson’s disease.

The pulsed ultrasound technique—essentially sonicating the BBB—allows for transient and repeated opening of the barrier, possibly allowing for diffusion of therapy into the brain.

The technique of pulsed ultrasound has been demonstrated in small animal models, and more recently in nonhuman primates. The effects of pulsed ultrasound can be monitored with MRI. It is associated with few side effects, such as red blood cell extravasation. It may result in minor damages to the brain parenchyma.

What are some limitations? ‘The skull presents a challenge,’ Dr Beccaria says. He stated that an ultrasound device has been developed that can be implanted into the skull, allowing repeated opening of the BBB.

Dr Beccaria said that the first clinical trial assessing BBB disruption with an implantable ultrasound inducer in glioblastoma patients is currently underway.

Dr Dannis van Vuurden returned to the podium to present on nanotechnologies and molecular drug imaging in DIPG and paediatric high-grade glioma.

On the topic of nanotechnology, he discussed the use of image-guided microbubbles to temporarily disrupt the BBB (in specific regions of the brain where the tumour resides) through sonoporation. Combining brain-targeted engineered liposomal drugs (which can cross the BBB) with microbubble-assisted delivery is hoped to result in higher intratumoral drug levels.

Molecular drug imaging (using radio-isotope-labeled drugs with PET scanning) will allow the measurement of drug uptake in tumours, and may help to identify which patients have the highest chance of benefit from these treatments (and prevent patients from getting drugs that do not, or ineffectively, reach their tumours).

Why does WNT-medulloblastoma have a good response to treatment, while other subtypes have such grim outlooks? Professor Richard Gilbertson of Cambridge University, Cambridge, UK discussed an interesting correlation between medulloblastoma genotype and BBB phenotype.

WNT-medulloblastoma is associated with aberrant ‘peripheralised’ BBB vasculature. This allows the accumulation of chemotherapy, leading to a robust therapeutic response. By contrast, SHH-medulloblastoma, which has a poorer outlook, is connected to an intact BBB that allows the tumour to resist chemotherapy.

The relationship between the tumour and the endothelial cells of the BBB can be manipulated *in vivo*, allowing for chemotherapy to penetrate the BBB. This suggests an approach to enhance the chemoresponsiveness of other medulloblastoma subtypes.

Dr Karin Straathof of the University College London Institute for Child Health, London, UK presented on the exciting topic of chimaeric antigen receptors (CARs). T-cells, white blood cells that are part of the immune system, can be paired with tumour-specific CARs to selectively target and destroy cancer cells. These CAR T-cells have shown great effectiveness in central nervous system leukaemia, and are an attractive new treatment modality. Dr Straathof turned her attention to their possible utility in intracranial tumours. Since T-cells can migrate into the CNS, this treatment bypasses the BBB, which Dr Straathof confirmed in animal models.

‘The first challenge of CARs in children is identifying new target antigens,’ Dr Straathof said. Her team and others are using bioinformatics approaches to identify candidate antigens with selective expression on the tumour, with the hopes of designing CAR T-cell therapies.

Dr Jordan Green of Johns Hopkins University, Baltimore, USA described an interesting novel approach to nanoparticle delivery, involving a type of biodegradable plastic (PBAE) that can form nanoparticles. These nanoparticles can be used to deliver drugs, or nucleic acids that reprogramme cancer cells ‘from the inside out,’ also known as ‘Suicide Gene Therapy.’ Dr Green hopes that nanoparticles will be able to hit previously ‘undruggable’ targets, delivering targeted or personalised therapy.

Previous attempts to delivery Suicide Gene Therapy centred around viruses as the delivery mechanism, but Dr Green suggests that a water soluble, nontoxic biodegradable polymer will be safer than viruses. Polymers are usually less effective than viruses, but their positive charge means that they self-assemble with nucleic acids, which makes them an attractive delivery material to form nanoparticles. Through a polymer library approach, Dr Green’s lab has engineered polymers particularly effective for delivery to brain tumours. Each particle can deliver multiple plasmids. After an infusion through convection-enhanced delivery in a glioma model, labelled nanoparticles were found to have penetrated throughout the tumour. PBAE nanoparticles loaded with siRNAs were found to efficiently deliver their load in human brain cancer cells cultured *in vitro*, sparing healthy human cells. Nanoparticles loaded with miRNAs were delivered to human brain tumour initiating cells, resulting in a reduction in neurosphere formation and reduced expression of stem cell markers.

Dr Green concludes that PBAE nanoparticles will be a safe and effective treatment for brain cancer.

## Interstitial delivery

The last session of the workshop focused on interstitial delivery. Dr Stuart Smith of the University of Nottingham, Nottingham, UK examined the lessons learned from Gliadel in practise and in clinical trials. Previous talks on this treatment provided a thorough grounding in the use and benefits of Gliadel, as well as its limitations. In his talk, Dr Smith focused on the limited clinical uptake of Gliadel in the paediatric setting, particularly in the UK, and some of reasons for this.

Previous speakers, including Dr Brem, had noted the common concern about infection at the surgical site. Although other speakers had suggested that this risk was found to be low, Dr Smith noted that this concern may have limited the uptake in paediatric settings. Other causes for concern include intracranial hypertension and wound leak, although Dr Smith says the incidence of these complications appears to be low. Gliadel remains one of very few agents to have shown real benefit in a phase III trial, and is one of only 3 pharmacological approaches funded and licensed for UK patients for Glioblastoma. One concern about Gliadel was the argument that there might be differences in the makeup of the wafers in the trial, although a Cochrane review found that the overall quality of the wafers was consistent and reliable. He noted, however, that Gliadel has little benefit for patients with diffuse CNS tumours. It is also limited in its inability to address distant and metastatic recurrence, but provides proof of concept for local drug delivery to tumours that recur at the surgical margin.

In Dr Smith’s experience, the paediatric outlook is promising—he noted that a current UK child patient is doing particularly well with Gliadel treatment. Its success also suggests further ways to exploit the intracavity treatment approach, and ways to build upon this success.

One method could be to decrease the initial ‘burst’ of the drug from the implanted wafer, which would allow the wound some time to heal. Dr Smith suggested that another promising delivery method could be filling the excised cavity with a drug-impregnated material such as foam, rather than lining it with flat wafers, as this could increase the life of the ‘implant.’ He also foreshadowed Dr Rahman’s talk to come, and noted that all of these emerging techniques paves the way for other novel agents to be developed and worked into the intracavity delivery paradigm.

Dr Ruman Rahman, also of the University of Nottingham, followed on from this by introducing a novel complementary drug delivery technique for interstitial administration, which builds upon these successes and overcomes some of the limitations of the Gliadel wafer. He introduced his group’s novel development of a mouldable polymer paste, which is loaded with chemotherapy and applied to cover the entire surface area of the resection cavity. The paste is a compound of poly(lactic-co-glycolic acid)/poly(ethylene glycol) microparticles, referred to as PLGA/PEG, and the chemotherapeutic agent, such as temozolomide. Dr Rahman compared its consistency during application to that of toothpaste; ten minutes after exposure to the patient’s body heat, the compound ‘sets’ and release of the drug begins. The PLGA/PEG system is biodegradable and has been shown to increase tumour necrosis and increase survival in rodents.

Next, Dr Rahman said, ‘we layered the nano on top of the micro.’ While PLGA/PEG paste is made of microparticles, his next development was a direct corollary to this system using *nano*particles. The group coated iron oxide magnetic nanoparticles with genetically-engineered peptides that induce membrane docking, cell penetration and cytotoxicity. When glioma cells were transduced with these nanoparticles, they showed truly impressive uptake (100% of cells after three hours’ exposure) and cytotoxic effects. This nanoparticle system can be combined with polymer paste to create a local delivery system. To the group’s knowledge, this is the first incorporation of therapeutic nanoparticles within local drug delivery depot.

If *in vivo* results continue to show promise, Dr Rahman aired the attractive possibility that these novel delivery systems could be used to repurpose previously developed drugs that failed to bypass the blood–brain barrier, or which previously showed intolerable systemic toxicity.

Dr John-Paul Kilday of of the Royal Manchester Children’s Hospital and Institute of Cancer Sciences, The University of Manchester discussed the use of intracystic therapies in paediatric craniopharyngioma. These tumours account for 5–10% of paediatric brain tumours. Classified as benign or Grade 1 lesions by the World Health Organisation, they can nonetheless be very damaging as their location is intimate to crucial CNS structures. Surgery is the best treatment, but has the risk of recurrence. Further, the location of hypothalamo-pituitary lesions proves a particular surgical challenge, and a risk of morbidity and mortality.

Conformal radiotherapy is the standard adjuvant treatment for craniopharyngioma, but as other speakers had noted, concerns about its effect on child development are critical. Some reports have suggested that aspiration of the cyst shows promising results. Dr Kilday stated that recent promise has been shown in intra-lesional or intracystic therapies.

Intralesional radioisotopes are limited in their accessibility depending on country, and there is little consensus between countries on a standardised practise. Bleomycin was put forth as a possible intracystic treatment, studied *in vivo* and *in vitro*, but Dr Kilday noted that a Cochrane review could not recommend its use. Thus, Dr Kilday focused his talk particularly on intracystic interferon-alpha therapy.

Intracystic interferon seems well tolerated, with Dr Kilday referencing a limited Latin American study by Cavalheiro *et al*. However, data on progression-free survival after its use was lacking. To address this, Dr Kilday has performed a global retrospective assessment of intracystic interferon use in children with craniopharyngioma across SIOP Europe and ISPN centres. Provisional data from this analysis was presented. Generally, this treatment seems to improve progression-free survival, and about half of patients had no side effects. However, Dr Kilday noted that these preliminary findings are the result of very small studies, and randomised clinical trials are needed to investigate further. Dr Kilday expressed his hopes that his presentation will inspire future collaboration and prospective clinical trials.

Professor Henry Brem delivered a closing lecture on recent developments in interstitial delivery. He mentioned that his work on Gliadel may have had unintended side effects for innovation. ‘Gliadel froze the technology,’ he said, ‘Once you get one polymer approved, you freeze [innovation].’ Likewise, he said, ‘we shouldn’t be talking, in 2016, about BCNU or temodor—there should be new agents to discuss.’

But there are some interesting advancements in the field. Polymer development may mean that drugs found to be effective in other cancers could now be delivered to brain tumours. Rapamycin, an immunosuppressive, anti-proliferative mTor inhibitor, is more effective when delivered in polymer than systematically. Anti-angiogenesis drugs are an attractive choice—although Professor Brem notes that shutting down angiogenesis means that one limits the ability of the bloodstream to transport and deliver drugs. Clinical trials of Taxol in combination with various polymers have shown promise.

Biodegradable microchips, which can be engineered to deliver a controlled release of multiple therapeutic agents at predetermined times, are an innovation to look out for. By releasing controlled amounts in multiphasic patterns, these microchips can more closely replicate physiological conditions. Microchips can be used to deliver combinations of agents, which Professor Brem expects to herald a new era of innovation in targeted drug delivery in brain tumours.

‘If you’re trying to juice up the immune system to fight cancer, does chemotherapy help or harm this cause?’ Professor Brem asked rhetorically. Chemotherapy is immunosuppressive, presenting a challenge for effective combination with immunotherapy. Professor Brem explained a study in which the combination of chemotherapy and targeted antibodies with anti-PD-1 showed strong survival and immunologic benefit in glioma-bearing mice, when compared to mice that received chemotherapy and anti-PD-1. These findings suggest a future pathway of clinical management for brain tumours—a combined, targeted approach.

## Conclusion

At the end of the workshop, chairman Professor David Walker summoned the participants to reflect upon the discussions that had taken place, and to commit to a new spirit of collaborative enterprise. He asked participants to consider the proposed Hatch, Match, Despatch and Deliver drug development pipeline, and reminded participants that Children with Cancer UK will be calling for project grant applications for innovative projects focusing on the delivery of therapy. ‘The idea is that in five years’ time we’ll have more drug delivery systems and trials than we do now,’ he said.

Professor Brem’s discussion of the treatment landscape for brain tumours in 1984 brought home the impact that innovation has wrought in recent years. The median survival time for patients with brain tumours has doubled since he began his research career in this field.

Yet in comparison to other cancers, the treatment of brain and CNS cancers is particularly challenging. The great advances made in other areas of oncology—such as targeted therapy, immunotherapy, and innovative delivery systems—here meet with the obstruction of the blood–brain barrier. And in the case of children with cancer, the hard-and-heavy approach to treatment causes significant quality-of-life issues, such that a more nuanced treatment approach is called for.

Yet the treatment landscape is improving, with innovation across disciplines holding new promise for brain and CNS tumour treatment. We now have access to technological advances including 3D printing, nanoparticles, genomic profiling, 3D cell culture, and robot-assisted surgery. And, most importantly, we have unprecedented appreciation for cross-disciplinary collaborative research.

‘None of this would have happened without collaboration between countries, between academics,’ Professor Brem said in his closing remarks. ‘My impression is that opportunities have never been greater. There is light at the end of the tunnel.’
